# The perceptions of parents about the skin conditions of their children presenting with comorbid fungal skin infections in Francistown, Botswana

**DOI:** 10.4102/phcfm.v5i1.459

**Published:** 2013-05-06

**Authors:** Deciderius C. Ifebuzor, Langalibalele H. Mabuza, Nomsa H. Malete, Indiran Govender

**Affiliations:** 1 Kagisano/Molopo Sub-district, Ganyesa, South Africa; 2 Department of Family Medicine & Primary Health Care, University of Limpopo, South Africa

## Abstract

**Background:**

In 2006, about 50% of the children whose parents brought them to the Francistown City Council clinics in Botswana for consultation had fungal skin infections. Most of these parents did not include the fungal skin conditions in the list of presenting complaints.

**Objective:**

To explore the perceptions of the parents about the fungal skin conditions of their children.

**Method:**

Eight participants were purposefully selected amongst the Francistown City Council clinics. They were interviewed, using the same exploratory question: ‘How much do you know about this skin condition?’ The Setswana translation is: ‘*O itse go le kae ka bolwetsi jone jo jwa letlalo?*’ The interviews were held in the Setswana language and audiotaped. The recordings were transcribed verbatim and translated into English. The ideas that emerged were developed into themes through the ‘cut-and-paste’ method.

**Results:**

The following themes emerged: the skin condition was not well-understood, it was perceived to have multiple causes, it was known to be infectious, many home remedies were used to attempt to cure it, it was not serious enough to warrant consultation and it tends to recur.

**Conclusion:**

Parents who brought their children to the Francistown City Council clinics in Botswana with fungal skin infections (incidentally discovered by the health care practitioners) perceived the skin infections as normal and not serious enough to be mentioned in a consultation. It is recommended that health care practitioners proactively educate parents of children presenting with comorbid fungal skin infections.

## Abstrait

**Comment les parents perçoivent-ils les problèmes de peau de leurs enfants présentant une infection fongique de la peau comorbide à Francistown, au Botswana**

**Contexte:**

En 2006, environ 50% des enfants amenés par leurs parents dans des centres médicaux de la municipalité de Francistown au Botswana présentaient des infections fongiques de la peau. La plupart de ces parents n'incluaient pas ces problèmes de peau à la liste des affections pour lesquelles ils venaient consulter.

**Objectifs:**

Étudier comment les parents perçoivent les infections fongiques de la peau de leurs enfants.

**Méthode:**

Huit participants ont été délibérément choisis dans les centres médicaux de la municipalité de Francistown. Ils ont été interrogés, chacun se voyant poser la même question: « Que savez-vous de cette infection cutanée? » La traduction de cette question en Setswana est la suivante: « *O itse go le kae ka bolwetsi jone jo jwa letlalo?* ». Les entretiens ont été menés en Setswana et enregistrés. Les enregistrements ont été retranscrits mot à mot et traduits en anglais. Les idées qui en ont émergé ont été développées en thèmes par la méthode du « copier-coller ».

**Résultats:**

Les thèmes suivants ont émergé: le problème de peau n’était pas bien compris, il était perçu comme ayant des causes multiples, les parents savaient qu'il était contagieux, de nombreux remèdes maison étaient utilisés pour essayer d'y remédier, il n’était pas suffisamment grave pour justifier une consultation, et tendait à être récurrent.

**Conclusion:**

Les parents qui amenaient leurs enfants dans les centres médicaux de la municipalité de Francistown, au Botswana, qui présentaient des infections fongiques de la peau (découvertes accidentellement par les professionnels de la santé), percevaient les infections cutanées comme normales et pas suffisamment graves pour être mentionnées lors d'une consultation. Il est recommandé que les professionnels de la santé informent de manière proactive les parents des enfants présentant des infections fongiques de la peau comorbides.

## Introduction

### Background

Fungal skin disorders amongst children are commonly encountered by primary health practitioners worldwide. *Trichophyton rubrum* is the most common dermatophyte species and the most frequent cause of fungal skin infections (dermatophytosis) in humans worldwide.^1^ The estimated lifetime risk of acquiring a fungal skin infection is between 10% and 20%. Dermatophyte infections are classified according to the affected body site: tinea capitis (scalp), tinea barbae (beard area), tinea corporis (body), tinea cruris (groin and perineum) tinea pedis (feet), tinea manuum (hands), and tinea unguium (nails).^2^ According to a recent survey in England and Wales, the incidence of new episodes of skin disorders (24%) exceeded incidences of all other major disease groupings, making them the most frequent reason for consultation in general practice.^3^ In South Africa, skin conditions were the second commonest presenting complaints amongst children under five years (23%) in two provinces (KwaZulu-Natal and Limpopo).^4^ At the time of the study, dermatophytosis was a major health problem amongst children in Botswana. The commonest clinically diagnosed dermatophyte was tinea capitis (scalp). Pityriasis versicolor, which is not a dermatophyte infection, was not seen in children – it occurred in adolescents and adults. Tinea capitis was also common in a study conducted in Dar es Salam, Tanzania.^5^ From our experience, more than 50% of children brought to the government clinic by their parents or guardians were infected.^6^ The prevalence was higher in rural areas due to poverty and low socio-economic conditions.^7^

### Aim of the study

It was not clear to the researchers why the parents did not include the fungal skin infections as a health care problem when bringing their children for other health problems. Did they regard it as a normal occurrence? If it was regarded abnormal, were there remedies other than Western medicine that they used for the condition? What did they think caused the skin conditions? To our knowledge, there was no published study in Botswana on the perceptions of parents of children with fungal skin infections about these conditions. The aim of the study was to explore the perceptions of parents about the skin conditions of their children presenting with comorbid fungal skin infections in Francistown, Botswana.

### Contribution to field

In this study, participants perceived dermatophytes in children as a normal childhood condition. This study highlighted the need to educate parents about the condition, as well as the need for every clinician to view encounters with patients as opportunities for health promotion and education.^8^

## Ethical considerations

Ethical approval for this study was obtained from the Research, Ethics and Publications Committee (ethics clearance number: MP 36/2006) and the Francistown City Council district. Standard ethical principles of autonomy, beneficence and justice were upheld throughout the study.

Allowing enough time for each respondent to exhaust an issue under discussion ensured validity. To prevent thwarting a respondent's thought process he or she was not interrupted with new questions whilst talking. In this way, the respondent had the opportunity to discuss an issue exhaustively. Only clarification questions were asked to ensure understanding of the issues under discussion. Feeding the information obtained back to the participants further ensured validity. Asking each respondent the same exploratory question and probing questions ensured reliability. Reliability was further ensured by the use of audiotape to preserve the collected data.

Purposeful sampling introduces sampling bias to a study. However, since we were looking for depth of information, this bias did not affect the study negatively. However, the result obtained is only be transferrable to other settings and cannot be generalised. Medical service delivery is free in public health care centres in Botswana. The fact that Francistown City Council clinics are public health care centres implied that private patients were excluded from the study, leading to selection bias.

## Method

### Design

The aim of the study was to explore the help-seeking behaviour of parents whose children had skin fungal infections. A qualitative study was conducted using the free attitude interview technique.

### Sampling and setting

The population comprised of all parents or guardians who attended the Francistown City Council clinics in Botswana. According to the clinic records, the average number of parents or guardians bringing children to the clinic for various conditions (including gastro-intestinal and respiratory infections) were 20 per day, of whom about two had dermatological problems. They brought their children aged between 5 and 15 years for various health problems, but excluded the skin infections from the list of presenting complaints. The clinician in attendance diagnosed the tinea clinically. Eight consented participants were purposefully sampled. Parents or guardians who resided in Francistown and who could communicate in the local language (Setswana) or English were included. Those who brought in very ill children and/or declined participation were excluded.

### Procedure

The principal investigator conducted face-to-face interviews for each participant. The exploratory question was: ‘How much do you know about this skin condition?’ (Translated into Setswana: ‘*O itse go le kae ka bolwetsi jone jo jwa letlalo?*’). An interview guide was used to supplement information on certain issues raised. For example: ‘Why did you not include it as one of the child's health problems?’, ‘Do you think the skin condition is treatable and how?’, ‘What do you think is the cause of the skin problem?’, ‘Is the skin condition infectious to other children?’ Interviews continued until saturation of data was reached.

The baseline characteristics of the participants (age, sex, occupation, educational background, marital status and monthly family income) were recorded for each participant. The interviews were conducted by the research assistance (a nurse proficient in English and Setswana who had undergone training in qualitative data collection) in either English or Setswana. The interviews were audiotaped and transcribed verbatim by a hired typist. Transcription was followed by the translation of the Setswana transcripts into English by a linguist expert. Back translation was performed from Setswana to English to ensure correctness of the translation.

### Analysing

A thematic data analysis was carried out: the transcripts were read several times to ensure content understanding. Similar ideas were grouped together into themes using the ‘cut-and-paste’ method. A combined list of themes from all eight interviews was compiled. A model was finally developed to represent a visual depiction of the interconnectedness of the themes.

## Results

### Participants’ characteristics

There were eight participants of whom five were grandmothers, two were mothers and one was a guardian of the child. The majority of the participants had primary education and the rest had no formal education. All of them were unemployed. All the participants were from a poor socio-economic class (class 5).

### Themes

#### Theme 1: Skin disease was not well-understood

Participants did not have the Western medical knowledge about the skin condition. The rash was not viewed as serious:

‘[*Yes*], I always see it on the bodies of the children, but I do not know anything about it.’ (BM, female, 77)

‘I do not know [*what it is*], but every child has it and we do not see it as a problem. I also have it on my body, but it is not causing any problems [*for*] me.’ (TN, female, 23)

They indicated that the skin condition was a common occurrence in children, as well as in adults:

‘I do not know much about it … All I can say is that in my house we all have it and we do not see it as a problem. At time[*s*] it will come and disappear.’ (YN, female, 67)

‘Y[*es*], I always see it on the bodies of the children and adults - we live with it. It is called [*a*] skin rash and at times it can itch or cause the body to be scratched.’ (CS, female, 40)

They described it simply as *Bogwata* - a Setswana term for any body rash:

‘It has no name. We call it Bogwata [skin rash].’ (SM, female, 77)

#### Theme 2: The skin condition was known to be infectious

The respondents observed that it was infectious, not only amongst the children, but that it could infect adults too:

‘… [*A*]ccording to my knowledge it is infectious to everybody, even to the children and nowadays it affects everybody.’ (BN, female, 70)

‘Yes, it is infectious to the children if they do not take their bath.’ (ES, female, 82)

‘I think it is infectious to children when they share clothes, or when they sleep together, or when they do not wash their body, and also when they sweat a lot.’ (PL, female, 88)

#### Theme 3: The skin condition was thought to have multiple causes

Participants thought that bad hygiene, allergies, HIV, ‘dirty’ blood, or the food they ate caused the skin condition:

‘I think [*the*] person [*is*] not taking care of him[*self*] well. Allergy, dirt … not taking a bath and what we eat these days [*cause it*]. It can also be from bad blood.’ (YN, female, 67)

‘If you do not take your bath you can also have it and it will be itching. Allergy [*and*] dirtiness can also cause it.’ (ES, female, 82)

‘I also heard that HIV causes it. My child has the virus and I think it is the virus that is causing it. The brothers at home also have the skin rash.’ (BM, female, 77)

‘These days they said it is also caused by HIV-infection.’ (CS, female, 40)

‘Dirt and not washing [*your*] clothes can cause it.’ (SM, female, 77)

‘When the blood is sick and not good.’ (BN, female, 70)

‘I think it is the food we eat these days [*that causes it*] …’ (TN, female, 23)

‘From my own knowledge, it is caused by not taking a bath, dirty environment, germs, allergy and some food we eat.’ (PL, female, 88)

#### Theme 4: The skin condition was viewed to be mainly treatable by home remedies

Participants reported that the skin condition was treatable with home remedies and traditional medicine:

‘I know that it is treatable. We treat it with “potash” [*potassium permanganate*], crushed charcoal with oil and some herbs from the bush.’ (CS, female, 40)

‘We have ways of treating it at home without coming to the clinic for it.’ (CS, female, 40)

‘I think it is treatable with calamine lotion from the clinic, brake fluid, and some herbs from the native doctor.’ (YN, female, 67)

However, treatment was warranted only if it was troublesome (symptomatic):

‘We only treat it if it is troubling the child and also when it is itching.’ (ES, female, 82)

‘My grandmother is a traditional doctor and she gives us medicine for it when it is itching or when it is troubling the child, so there is no need telling the doctor about it.’ (TN, female, 23)

#### Theme 5: The skin condition was not serious enough to be presented for treatment at the clinic

In explaining why the skin condition was not included in the list of presenting complaints for the current presentation, the parents or guardians indicated that they viewed it as normal for children to develop it in their childhood:

‘It is normal in children at this age.’ (BN, female, 70)

‘At this age children change colors on the skin and when they grow up, the good color comes out.’ (PL, female, 88)

If it was not troubling the child, there was no need for them to complain about it to the health care professional:

‘I believe it is not a problem and my sister is not complaining about it. My mother has medicine at home for treating it. So we can treat it at home, not in the clinic.’ (SM, female, 77)

‘Because it is not troubling the child and the child is not complaining about it. It is not itching and we also have medicine at home for treatment. It is not a serious problem.’ (BM, female, 77)

#### Theme 6: The skin condition tended to recur after treatment

The participants admitted that it was not easily treatable with the home remedies. One participant indicated that she was tired of presenting the skin condition at the clinic, because it would recur after treatment:

‘Another thing I want to say is that it is difficult to treat these days. After treating it, it will come back again. So I feel there is no need complaining about it to the doctor.’ (CS, female, 40)

‘Another thing is that it is difficult to treat … After treating it, it will come back again, so I am tired of complaining since it is not disappearing completely …’ (YN, female, 67)

**FIGURE 1:**
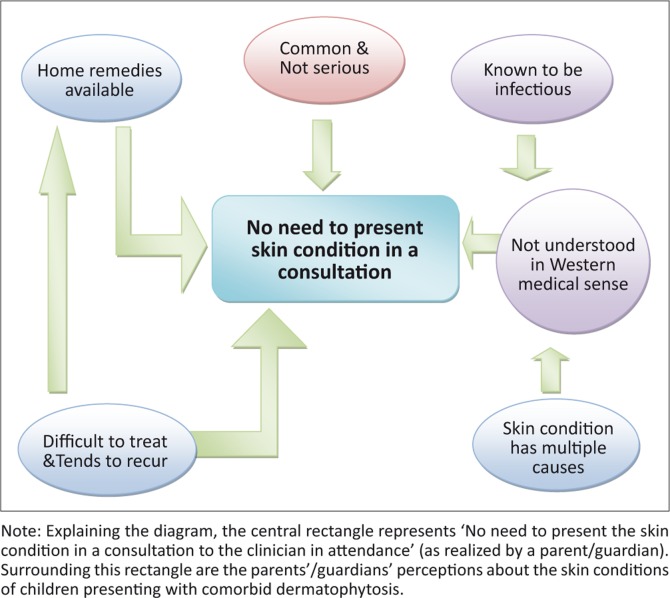
Model of parents’ perceptions of fungal skin conditions.

The illustration presented in Figure 1 is a model of parents’ perceptions of fungal skin conditions illustrating the relationship of the themes.

## Discussion

The study has demonstrated that, primarily, the parents of children with fungal skin conditions did not understand the condition in the Western medical sense. They perceived the fungal skin conditions as a normal common occurrence in children in their community. There was no specific name in Setswana for the skin conditions – they were simply referred to as *Bogwata* (a general Setswana term for any body rash). It was not perceived serious enough to be mentioned in a consultation about other health problems. The parents were aware that the skin condition was contagious and that it was associated with poor hygiene. The parents has a number of home remedies for the condition, but noticed that the condition tends to recur after using those remedies.

The parents had a general term for the fungal skin conditions. The problem with that generalisation is that there are many fungal skin conditions that differ widely and they are not treated in the same way. For example, tinea capitis is treated systemically with griseofulvin (8 weeks) or terbinafine (4 weeks) and does not respond, for example, to selenium sulphide used in the treatment of tinea vesicolor.^9^ Knowledge on the various fungal skin condition types and their management would result in appropriate health-seeking behaviour from these parents to the benefit of their children. Our study showed that the skin conditions did not cause any anxiety or intolerance amongst the parents. This is in keeping with the notion that a patient will seek medical attention when they reach their limit of tolerance and/or anxiety about the condition they suffer from.^10^

The skin conditions were not perceived serious enough to be included in the list of presenting complaints in the current consultation. It was viewed as a normal part of childhood as the condition would be corrected spontaneously as the children grew older – they would revert to ‘the good colour’. Heukelbach et al. had similar findings on the health-seeking behaviour of patients who presented with parasitic skin infections in the Northeastern Brazilian community. Their study found that patients neglected and did not consult for their parasitic skin infestations, which impacted on the true prevalence of the diseases in that community.11 The fact that it was not viewed seriously could result in complications. Tinea capitis has the potential to cause scarring and permanent alopecia, whilst tinea corporis can lead to secondary bacterial infections and permanent changes in skin colour.^12^

In our study, the participants reported that they were not so keen to present it again in subsequent visits since the skin conditions tended to recur even after the clinic's treatment. The recurrence could be explained at various levels: clinic factors (for example the unavailability of medication), patient factors (for example non-compliance to medication given), health care practitioner factors (for example lack of health care education) and promotion, including education on proper hygiene. Looking at the health-seeking behaviour of patients with Buruli ulcer in southern Benin, Aujoulat et al. also found that patients tended to be reluctant to seek medical attention at health care centres if the available treatment was considered ineffective.13

We found that the parents noticed that the skin conditions were infectious – not only amongst the children, but also amongst adults. The causes of the skin conditions were perceived as multiple, including bad hygiene, allergy, HIV, the food they ate, ‘bad blood’ and the ‘dirty environment’. The fact that the parents mentioned HIV was not far-fetched, since a study in Tanzania showed the association of immunosuppression with fungal skin infections, especially in the advanced stages of HIV.14 However, in our study not all the children with fungal skin conditions appeared to be in the advanced stages of HIV. The skin conditions were viewed treatable mainly by home remedies, including ‘potash’ (potassium permangate), crushed charcoal mixed with oil, brake fluid and herbs. Calamine lotion, which is not a home remedy but western medication, was also mentioned as treatment. A study by Doulgeraki and Valari in Greece found that 38% of parents used products containing natural oils, whilst almost half of the parents applied botanical and commercially available synthetic products in the prevention of head lice for their children.^15^ Since calamine lotion is not an anti-fungal remedy, the health care practitioners may have dispensed it as remedy for other conditions the patients had presented with in the past, or it may have been dispensed for its antipruritic effect against itchy skin conditions.16 The application of brake fluid on the skin for medicinal use is not recommended as some types of hydraulic fluids can irritate the skin or eyes.^17^

The participants did not know that the skin conditions were infections caused by microorganisms (except for one who referred to ‘a germ’ as the cause). The lack of scientific knowledge of the cause of the skin conditions explained why the parents mentioned remedies that have not been scientifically proven to be helpful to treat the condition. The children would continue to suffer from the conditions as long as their parents held the unscientific perception. The health care practitioners could reinforce the parents’ knowledge that the skin conditions are infectious by promoting good hygiene to prevent their spread. Some of the parents mentioned that ‘not taking a bath’ caused the condition. Although bad hygiene per se is not a cause of the condition, good hygiene generally prevents the spread of infections, including fungal infections.^18^

### Limitations and strengths of the study

The study investigated a phenomenon that is frequently encountered at the primary health care level, but that has not been previously explored in this setting. The qualitative approach enabled exploration of the depth of information. The limitation is that the findings of a qualitative study cannot be generalised.

### Recommendations

It is recommended that health care practitioners proactively educate parents and guardians of children presenting with comorbid fungal skin infections to inform them that the skin conditions are not normal and that they are amenable to medical treatment.

## Conclusion

Parents who brought their children with fungal skin infections (incidentally discovered by the health care practitioners) to the Francistown City Council clinics in Botswana perceived the skin infections as normal and not serious enough to be mentioned in a consultation. They perceived the skin conditions as having multiple causes, but none of those mentioned were science-based. To that end, they used remedies that have not been proven scientifically. It is recommended that health care practitioners proactively educate parents presenting with comorbid fungal skin infections to inform them that they are abnormal and should be treated with appropriate medication.
